# Health Effects of Naturally Radioactive Water Ingestion: The Need for Enhanced Studies

**DOI:** 10.1289/ehp.1003224

**Published:** 2011-08-02

**Authors:** Irina Guseva Canu, Olivier Laurent, Nathalie Pires, Dominique Laurier, Isabelle Dublineau

**Affiliations:** 1 Service de Radiobiologie et Epidémiologie, and; 2 Service d’Etudes et Expertise en Radioprotection, Institut de Radioprotection et de Sûreté Nucléaire, Fontenay-aux-Roses, France

**Keywords:** Drinking water, epidemiology, ingestion, natural radioactivity, toxicity

## Abstract

Background: Radiological pollution is a potentially important aspect of water quality. However, relatively few studies have been conducted to document its possible health effects.

Objective: In this commentary we discuss available epidemiological findings and related data from experimental studies concerning the health effects of naturally radioactive water ingestion.

Discussion: Despite modest epidemiological evidence of uranium nephrotoxicity and radium effects on bone, available data are not sufficient to quantify the health effects of naturally occurring radionuclides in water. Methodological limitations (exposure measurement methods, control for confounding, sample size) affect most studies. Power calculations should be conducted before launching new epidemiological studies focusing on late pathological outcomes. Studies based on biomarkers of exposure and adverse effects may be helpful but should involve more specific molecules than biomarkers used in previous studies. Experimental data on ingestion of drinking water are limited to uranium studies, and there is some disagreement between these studies about the nephrotoxicity threshold.

Conclusion: Further experimental and enhanced epidemiological studies should help to reduce uncertainties resulting from dose estimation to dose–response characterization.

Radiation exposure through drinking water results from naturally occurring radionuclides in drinking water sources, in particular alpha-radiation–emitting uranium, radium, and their progeny, including radon. According to the World Health Organization (WHO), when activity concentration in drinking water exceeds the recommended level of 0.5 Bq/L for gross-〈 or 1 Bq/L for gross-® activities [simultaneously measured activity from a mixture of natural alpha [uranium-238 (^238^U), ^234^U, thorium-232 (^232^Th), radium-226 (^226^Ra), and polonium-210 (^210^Po)] and beta emitters [^228^Ra and lead-210 (^210^Pb)], radionuclide-specific concentrations should be brought into compliance with WHO guidance levels: 0.1 Bq/L for ^228^Ra; 1 Bq/L for ^223–226^Ra, ^234^U, and ^235^U; 10 Bq/L for ^238^U; 100Bq/L for radon-222 (^222^Rn), and 15 µg/L for total uranium (WHO 2004).

Ingested radionuclides are absorbed into the blood (International Commission on Radiological Protection 2007) and accumulate in specific tissues that they may damage. Of absorbed uranium, 66% is rapidly eliminated via urine, while the rest is distributed and stored in the kidney (12–25%), bone (10–15%), and soft tissue (Wrenn et al. 1985). Radium deposits mostly in the bone (Wrenn et al. 1985). Ingested radon gas diffuses into the stomach wall, making the stomach wall the tissue most irradiated by ingested radon because of its short half-life (3.8 days) (Hopke et al. 2000).

Natural uranium induces chemical toxicity, especially nephrotoxicity, which is more harmful than radiotoxicity; whereas radium and radon are thought to induce solely radiotoxicity (Wrenn et al. 1985).

Although some epidemiological studies have addressed the question of the possible health effects after ingesting naturally occurring radionuclides through drinking water (Kurttio et al. 2002, 2006a; Mao et al. 1995; Selden et al. 2009), their results have not been summarized to date. In this commentary we discuss available epidemiological findings and evidence of possible biological effects.

## Synopsis of the Available Epidemiological Evidence

We searched the databases PubMed (http://www.ncbi.nlm.nih.gov/pubmed) and Scopus (http://www.info.scopus.com) to identify all epidemiological studies dealing with potential health effects of naturally occurring radionuclides in drinking water reported for 1970–2009. For search terms, we used combinations of the key words “health,” “water,” and “radioactivity.” The word “health” was alternatively replaced by “epidemiology,” “case control,” “cohort,” and “cancer.” The word “radioactivity” was alternatively replaced by the names of the elements occurring in the decay chains of interest, namely uranium, thorium, protactinium, actinium, polonium, bismuth, radon, thallium, and lead. References in each paper were reviewed for additional sources. Only relevant articles published in English in peer-reviewed journals were retained. We identified 27 peer-reviewed published reports of original epidemiological studies, including studies of uranium, radium, and radon (Table 1).

**Table 1:** Available epidemiological studies on the possible effects of naturally occurring radionuclides in drinking water.

Study	Design	Radionuclide	Average concentration in water	Outcome	No. of subjects
Mao et al. 1995*		Cross-sectional		U		19.6 µg/L		Biomarkers of renal (glomerular) damage		140 cases
Zamora et al. 1998*		Cross-sectional		U		100 µg/L		Biomarkers of renal (tubular) damage		50 cases
Kurttio et al. 2002*		Cross-sectional		U		131 µg/L		Biomarkers of renal (tubular) damage		325 cases
Kurttio et al. 2005*		Cross-sectional		U		124 µg/L		Biomarkers of renal (tubular) damage		288 cases
Kurttio et al. 2006a		Cross-sectional		U		25 µg/L		Biomarkers of renal (tubular) damage		193 cases
Selden et al. 2009*		Cross-sectional		U		180 µg/L		Biomarkers of renal (tubular) damage		454 cases
Zamora et al. 2009*		Cross-sectional		U		88 µg/L		Biomarkers of renal (tubular) damage		54 cases
Petersen et al. 1966*		Ecological		^226^Ra		170 mBq/L		Bone cancer mortality		267 cases
Bean et al. 1982*		Ecological		^226^Ra		>110 mBq/L		Cancer incidence		1,596 cases
Lyman et al. 1985*		Ecological		^226^Ra		>185 mBq/L		Leukemia incidence and mortality		873 incident/890 mortality cases
Fuortes et al. 1990*		Ecological		^226^Ra		NR		Leukemia incidence		700 cases
Hess et al. 1983		Ecological		^226^Ra		NR		Cancer incidence		33,928 cases
Collman et al. 1988		Ecological		^222^Rn		NR		Cancer mortality		Total cancer cases NR (1,758 leukemias)
Collman et al. 1991*		Ecological		^222^Rn		NR		Cancer mortality		2,706 cases (1,194 leukemias)
Kjellberg and Wiseman 1995*		Ecological		^222^Rn		NR		Stomach cancer incidence and mortality		NR
Cech et al. 2007*		Ecological		^226^Ra		> 110 mBq/L		Orofacial cleft defect births		167 cases
Cech et al. 2008*		Ecological		^226^Ra		> 110 mBq/L		Orofacial cleft defect births		300 cases
Moss et al. 1995		Case–control		Gross α		300 mBq/L		Osteosarcoma incidence		167 cases/989 controls with other cancers, matched on age, sex, and race
Guse et al. 2002		Case–control		^226+228^Ra		NR		Osteosarcoma incidence		319 osteosarcoma cases/3,198 general population controls matched on age, sex, and ZIP code
Finkelstein 1994*		Case–control		^226^Ra		26 mBq/L		Bone cancer mortality		283 cases/285 controls (died of any other disease) matched on age, sex, and year of death
Finkelstein and Kreiger 1996*		Case–control		^226^Ra		26 mBq/L		Bone sarcoma incidence and mortality		583 cases/754 controls with (or died of) any other disease matched on age, sex, and year of death or diagnosis
Hirunwatthanakul et al. 2006*		Case–control		^226^Ra		NR		Digestive cancer incidence		32 cases/138 randomly selected healthy controls
Witmans et al. 2008*		Case–control		U		≈ 1 µg/L		Non-Hodgkin lymphoma incidence		88 cases/132 controls matched on age and sex
Seiler 2004		Case–control		U		≈ 2 g/L		Leukemia incidence		16 wells as cases/100 other community wells as controls
Auvinen et al. 2002		Case–cohort		U		0.45 Bq/L		Leukemia incidence		35 cases/274 controls matched on age and sex
				^226^Ra		30 mBq/L				
				^222^Rn		500 Bq/L				
Auvinen et al. 2005		Case–cohort		U		0.45 Bq/L		Stomach cancer incidence		107 cases/371 controls matched on age and sex
				^226^Ra		30 mBq/L				
				^222^Rn		500 Bq/L				
Kurttio et al. 2006b		Case–cohort		U		0.45 Bq/L		Urinary cancer incidence		112 cases (61 bladder, 51 kidney)/274 controls matched on age and sex
				^226^Ra		30 mBq/L				
				^222^Rn		500 Bq/L				
NR, not reported. *Statistically significant increase in the health damage of interest.										

Seven cross-sectional studies evaluated uranium in drinking water and individual biomarkers of chemotoxicity (urinary albumin, creatinine, glucose, phosphate, calcium, microglobulins, and enzymes). Overall, they reported associations between uranium concentrations in drinking water and indicators for cytotoxic damage to the proximal tubule of the kidney nephron (Selden et al. 2009; Zamora et al. 1998, 2009) and alteration of the renal absorption function (Kurttio et al. 2002, 2006a; Selden et al. 2009; Zamora et al. 1998, 2009). Another study reported a positive association with serum carboxy-terminal telopeptide, an indicator of bone resorption, in males (Kurttio et al. 2005).

When cumulative intake of uranium was estimated, null or nonsignificant associations with the studied biomarkers were found (Kurttio et al. 2002, 2006a; Mao et al. 1995). This may indicate that long-term uranium exposure through drinking water ingestion had no effect or that cumulative uranium intake based on self-administered questionnaires was not estimated accurately (Kurttio et al. 2002, 2006a). Inadequate control of confounding and insufficient power could also have been a problem.

Cancer was investigated in 10 ecological studies in the United States. These studies focused on the relationships between uranium, radium, or radon concentrations in drinking water (either in private wells or community supplies), averaged across counties or municipalities, and rates of cancer incidence or mortality measured at the same aggregation levels. In Iowa, Petersen et al. (1966) found that bone cancer mortality rates in people 20–29 and 60–69 years of age were significantly higher in towns with water supplies containing ^226^Ra concentration > 110 mBq/L compared with other towns. However, the outcome used for the study (deaths due in any way to malignant neoplasm involving bone, based on death certificates codes) did not rely on a standard definition for bone cancer. Bean et al. (1982) examined the incidence of various cancer sites in 28 Iowa towns, based on two national cancer survey programs. They found increased rates of bladder cancer in males, breast cancer in females, and lung cancer in both sexes in association with increasing ^226^Ra concentration in community water supplies. Bone cancer and leukemia were not studied as their rates were judged to be too unstable for analysis. In contrast, Lyman et al. (1985) specifically focused on leukemia incidence in 27 Florida counties. They reported a strong association with the percentage of samples from groundwater supplies that showed total radium concentration exceeding 185 mBq/L in these counties. Fuortes et al. (1990) observed a weak and nonsignificant association between leukemia incidence and ^226^Ra concentration in water in study participants in 59 Iowa towns.

Ecological studies on radon in drinking water exhibit broadly comparable patterns. Hess et al. (1983) analyzed cancer incidence in Maine counties (based on 1950–1969 National Cancer Institute statistics) and reported associations between average radon levels in water supplies and rates of all cancers combined as well as respiratory, testis, and prostate cancers in the counties. Collman et al. (1991) employed a similar approach in North Carolina and reported associations with all cancers and leukemia mortality in children; however, they observed no association in adults (Collman et al. 1988). Kjellberg and Wiseman (1995) specifically focused on stomach cancer incidence and mortality in Pennsylvania and significant associations were found, but their magnitude was not reported. No information about other risk factors for stomach cancer (e.g., food habits, smoking) was available.

Four case–control studies estimated associations between ingestion of radium via drinking water and bone cancer. Two studies were conducted in Wisconsin, based on the same cancer registry, over the periods 1979–1989 (Moss et al. 1995) and 1980–1997 (Guse et al. 2002); it is not clear whether any cases may have been included in both analyses. Moss et al. (1995) reported a positive but nonsignificant association between osteosarcoma incidence and gross-〈 activity exceeding 330 mBq/L in county water supplies, whereas Guse et al. (2002) reported no association with radium levels in drinking water supplies. Two case–control studies were conducted on bone cancer and ^226^Ra in water supplies at birthplace residences in Ontario, Canada, based on mortality (Finkelstein 1994) and incidence data (Finkelstein and Kreiger 1996). The first study reported a significant association between mortality for each subtype of bone cancers combined and ^226^Ra concentration in community water supplies or birthplace private wells. Both studies reported associations between osteosarcoma and ^226^Ra birthplace concentrations, and a significant association was reported based on a combined statistical analysis of the two studies (Finkelstein and Kreiger 1996). A significant association was also observed for all sarcomas. However, in this combined analysis, reconstituted (and partly simulated) lifetime ^226^Ra exposure estimates were not significantly associated with these outcomes.

A case–control study of digestive cancer incidence in Thailand reported an association with estimated oral radium consumption per day, but this was based on only 32 cases (Hirunwatthanakul et al. 2006).

In a Saskatchewan, Canada, case–control study on non-Hodgkin lymphoma incidence, cases had higher uranium concentrations in their drinking water than controls (Witmans et al. 2008). Seiler (2004), in a case–control study of 16 leukemia cases in Fallon, Nevada, found no significant differences in well uranium or radon concentration between cases and controls.

The only cohort study investigating the association between water radioactivity and cancer incidence is one of Finnish study subjects using bedrock well water. Authors used this cohort to conduct three case–cohort studies on 107 stomach cancer cases (Auvinen et al. 2005), 112 urinary cancer cases (Kurttio et al. 2006b), and 35 leukemia cases (Auvinen et al. 2002). No significant associations were reported, either with radionuclide concentrations (uranium, radium, and radon) in well water or with cumulative radiation doses when estimated (Kurttio et al. 2006b). However, each study included a relatively small number of cases and therefore had only modest statistical power.

Potential reproductive toxicity of radium and radon was studied in Harris County, Texas. Cech et al. (2007) reported that rates of orofacial clefts (based on birth certificates over the 1990–1994 period) were significantly higher in administrative areas (defined by postal code) with ^226^Ra concentrations exceeding 110 mBq/L than in areas with lower concentrations. Results were similar when the study was repeated in 1999–2002 using updated total radium measurements (Cech et al. 2008).

## Limitations and Uncertainties

Most reviewed studies on natural radioactivity in drinking water had important limitations with regard to exposure assessment, which can bias measures of association. First, dose assessment errors can result from sampling and analysis of water. In estimating retrospective cumulative intake, authors have assumed constant uranium concentrations in water over time, although these actually vary widely depending, for instance, on carbon dioxide partial pressures, pH of the source aquifer, and season (Ribera et al. 1996). Water radionuclides mitigation was considered in one study of uranium (Zamora et al. 1998) but not of radium, although most were based on public water supply measurements, and water softening is known to decrease radium concentrations substantially (Vesterbacka and Salonen 2008). The physicochemical nature of the contaminant, that is, determining its chemical speciation in water and biological fluids, was not accounted for. This might produce misleading results as some species (e.g., calcium-uranyl-carbonato complexes) are not cytotoxic, whereas others (e.g., uranium carbonate or citrate) are (Prat et al. 2009).

Second, few studies accounted for individual water consumption patterns (Auvinen et al. 2002; Hirunwatthanakul et al. 2006; Kurttio et al. 2006a), and few have considered individual residential mobility and changes in water supplies over time [e.g., via collection of individual residential histories (Finkelstein and Kreiger 1996)] although some specifically selected subjects who did not change residences (Auvinen et al. 2002; Kurttio et al. 2006b). Other studies characterized water quality at residential locations either at the time of diagnosis or death (Guse et al. 2002) or at study subjects’ birthplaces (Finkelstein 1994). Temporality of exposure assessment may also be an issue in cross-sectional studies of uranium chemotoxicity. Water and urine samples were obtained from participating individuals at the same time point, which would not allow judging whether exposure preceded effects.

A third and closely related issue is that most cumulative exposure assessment was based on cumulative duration of water consumption, whereas the period during which doses are delivered to target organs is a function of radionuclide intake and retention in these organs (i.e., radium or uranium sequestered in the bone). Inadequate accounting for the retention period may raise a problem of classification of the relevant dose and consequently of the dose–response magnitude. This is especially true when estimating the effects of exposures that occur during exposure windows of high sensitivity, such as fetal or child development, because of anatomical and physiological differences (e.g., higher gastrointestinal absorption of radionuclides, higher bone formation/resorption rate). Lack of information about individual differences in absorption and biokinetics could also lead to bias in the quantification of the relevant dose.

Finally, the latency period between exposure-related initiation and clinical diagnosis may be decades for some cancer but has not been accounted for (by lagging dose estimates) in most studies.

Only two studies (Hirunwatthanakul et al. 2006; Zamora et al. 1998) considered exposure to radionuclides from sources other than drinking water. However, the intake derived from food (mostly vegetables, fruit, and grains) can be > 80% for total uranium intake and about 50% for total radium intake (Wrenn et al. 1985). For radon, inhalation of airborne radon released from soils or water constitutes the major route of exposure (Hopke et al. 2000), and that particular route has not been considered in studies of this radionuclide. Further, coexposure to arsenic or terrestrial gamma rays, which may be higher in uranium- and radon-rich areas (Seiler 2004), was not considered in the reviewed studies. Collecting more data about individual lifestyle patterns (e.g., dietary patterns, smoking) would have been useful to assess potential confounding. For instance, except for Kurttio et al. (2006a, 2006b), smoking was rarely adjusted for in studies.

Most of the reviewed studies included modest (< 100) numbers of cases (especially for specific pathological subgroups (Auvinen et al. 2002; Seiler 2004), which limited their statistical power to detect and precisely quantify associations between health risks and radiation in drinking water. Both study sizes and duration of follow-up contributed to this limitation.

## Biological Effects and Action Mechanisms

The most relevant animal studies on chronic drinking water ingestion effects were performed for uranium. Biological effects of uranium in kidneys were reported, including modifications in renal metabolism of xenobiotics (Souidi et al. 2005), vitamin D homeostasis (Tissandie et al. 2007), and iron homeostasis (Berradi et al. 2008). Excessive iron accumulation and apoptosis in the tubulointerstitial region and uranium-induced oxidative stress were reported (Linares et al. 2006; Taulan et al. 2004). Histological lesions of renal tissue were also observed, mainly in the cortical part of the kidneys (Donnadieu-Claraz et al. 2007; Gilman et al. 1998; Ortega et al. 1989). Gilman et al. (1998) reported the lowest threshold of adverse effects for the kidney at 0.03 mg/kg/day, and this threshold was used to establish WHO guidelines for uranium in drinking water (WHO 2004). *In vitro* studies demonstrated that uranium alters the expression of genes involved in the cytochromes P450 and glutamate metabolic pathways, cell signaling and trafficking (Prat et al. 2010; Vietti and Lasley 2007), and deregulation of the apoptotic process (Prat et al. 2010). Experimental studies indicated that *in vivo* tissue effects of uranium in kidneys are not always reflected by modifications of renal and plasma parameters. A few authors have demonstrated modifications of plasma biochemical parameters, notably urea and creatinine, despite the absence of molecular or histological effects. This may partially explain (along with the methodological limitations of epidemiological studies discussed above) the modest evidence of renal uranium effects observed in human studies. This would argue in favor of launching epidemiological and experimental studies concurrently, using the most appropriate biomarkers of adverse effects. Regarding bone, uranium induces inhibition of osteoblastic activity, resulting in bone volume decrease and healing interference (Guglielmotti et al. 1987). *In vitro* studies showed that uranium induces genomic instability and neoplastic transformation in osteoblasts (Miller et al. 1998, 2003) and modifies oxidative metabolism and reduces bone formation (Tasat et al. 2007). According to Prat et al. (2010), uranium also alters the expression of the gene for osteopontin, a candidate biomarker of bone resorption and urolithiasis.

Concerning radium, most animal studies have investigated effects on bone after radium injection. Both ^226^Ra and ^228^Ra rapidly induce changes in bone structure and hematopoiesis. Bone sarcomas were found in all species tested within a life span follow-up [Agency for Toxic Substances and Disease Registry (ATSDR) 1990]. Leukemias were reported after ^224^Ra injection (ATSDR 1990). The major evidence concerning radium exposure effects come from epidemiological studies of radium dial painters (Rowland et al. 1978), which indicated a positive dose–response relationship in humans for bone and head and neck sarcomas. Authors concluded that ^228^Ra was about twice as effective at causing bone sarcoma as ^226^Ra, whereas head carcinoma was associated only with ^226^Ra. Radium dial painters were not at increased risk of leukemia, which was unexpected given that radium accumulation in bone would be expected to affect potentially leukemic bone marrow cells. Possible explanations include nonuniformity of irradiation, lethality in target cells, or a low frequency of susceptible target cells in irradiated regions. In addition, there was no conclusive evidence of increased risks of other cancers (Mays et al. 1985).

Experimental studies that reported effects of radon-contaminated drinking water are very rare (Masse et al. 1992; Sullivan et al. 1986). Because of this lack of data, large uncertainties remain concerning the transit time of radon throughout the gastrointestinal tract and whole-body radon retention. These uncertainties lead to dose overestimation by greater than two orders of magnitude, depending on the model used for dose calculation (Kendall and Smith 2002).

## Conclusion and Perspectives

Despite modest human epidemiological evidence of uranium nephrotoxicity and radium bone carcinogenicity, available studies do not clearly demonstrate the health effects of radionuclides at levels naturally encountered in drinking water. Methodological limitations (exposure assessment, possible confounders, limited sample size), affecting most reviewed studies, should be remedied in future studies.

New prospective cohorts, including potentially sensitive subpopulations (children, pregnant women) should be set in geographic regions known to have elevated concentration of radionuclides in drinking water sources (e.g., Finland and Canada for uranium, Iran and north-central states of America for radium). Exposure and biological effects assessments would be more informative if performed within the framework of prospective surveys and based on biochemical analyses of water (including chemical characterization of radionuclide contaminant) and individual biological samples (urine, blood). For uranium, urinalysis is a gold standard for exposure monitoring, whereas for radiotoxic contaminants, organ-absorbed doses must be assessed to estimate cumulative exposure. New experimental data are necessary to resolve uncertainties surrounding transit time of radon throughout the gastrointestinal tract and whole-body radon retention and thus improve precision of internal dose assessment. Measurement data should be coupled with questionnaires on water and diet consumption patterns. These questionnaires should also collect information on all potential risk factors for the health outcomes studied (e.g., smoking, occupational exposures). Residential mobility should be documented via active follow-up of individuals. To study cancer, residential exposures to radon and gamma rays should be measured in each subject’s residence.

Statistical power should be calculated before launching epidemiological studies on late multifactorial pathological outcomes such as cancers. In epidemiological studies looking at biomarkers of subclinical effects, such calculations are still feasible but might be less accurate because of a lack of data relevant to assumptions used to estimate power. Some biomarkers of early adverse effect could be more sensitive indicators than disease or death; therefore, their use in epidemiological studies would require fewer subjects. However, most currently available biomarkers tend to have low sensitivity and specificity as proxy measures of adverse effects. According to Adler (2010), even the biomarkers considered to be the best choices for diagnosing early kidney damage (i.e., *N*-acetyl-®-glucosaminidase, human-kidney-injury-molecule-1, cystatine-c, glutathione *S*-transferase) do not meet all the criteria of a desirable biomarker, especially in terms of specificity. However, simultaneous use of complementary biomarkers can be useful to compensate for their respective limitations.

Further research should be conducted to identify biomarkers with a better specificity and sensitivity for the contaminant and/or disease. The time scale of the biomarker, exposure, and outcome to get the best matchup of the exposure–dose and dose–response relationships must be considered. Research must also be developed to relate levels of these biomarkers to a probability of future disease occurrence in order to interpret results from studies based on biomarkers of early effects in a public health perspective.

Given the extent of these challenging tasks and the need for sufficient statistical power to detect potentially subtle effects (which might have non-negligible collective impacts in view of the large numbers of people exposed), sharing of means and expertise from several research teams would probably be necessary to conduct joint studies. Ideally, this would be completed within the framework of international collaborations.
